# Ambergris

**Published:** 1904-04

**Authors:** 


### AMBERGRIS.

The lump of ambergris stolen by two thieves in California the other day, and subsequently recovered, is said to have been worth $30,000, and was doubtless one of the largest pieces of that substance ever known. Certainly the finest, and perhaps the biggest, chunk of it obtained in modern times was sold in London in 1891. It weighed 163 pounds, and fetched $50,000.

There is no longer any mystery as to the origin of ambergris. It is a morbid secretion due to a disease of the liver of the sperm whale, in the intestines of which animal lumps of it are occasionally, though rarely, discovered. Dr. C. H. Stevenson, of the United States Fish Commission, who has made a special study of the subject, says that the whales which yield ambergris are invariably sickly and emaciated.

Anciently, the substance was known as amber—a name which was subsequently applied also to the fossil gum now commonly so called. But, to distinguish the two, one was called "amber gris" (gray), and the other "amber jaune" (yellow). So it appears that ambergris means simply gray amber. Like the fossil gum, pieces of it were found now and then on the seashore, where they had been cast up by the waves; hence, doubtless, the giving of the same name to both.

Doctor Stevenson says that ambergris usually contains the beaks of cuttlefishes, on which the sperm whale feeds. Sometimes it is black, but the finest is gray in color. When dried, to cure it, it is light and inflammable, and yields an odor faintly resembling honey. On being melted by heat it evaporates slowly, leaving no trace behind.

The substance has been used for centuries in sacerdotal rites of the church, and with fragrant gums was formerly burnt in the apartments of royalty. To some extent it was employed as a medicine and to flavor certain dishes. Nowadays it is utilized almost exclusively by perfumers, in the preparation of fine scents, being first converted into a tincture by dissolving it in alcohol.—*Saturday Evening Post.*

